# Associated Determinants Between Evidence of *Burnout*, Physical Activity, and Health Behaviors of University Students

**DOI:** 10.3389/fspor.2021.733309

**Published:** 2021-10-20

**Authors:** Rafael Octaviano de Souza, Flavio Ricardo Guilherme, Rui Gonçalves Marques Elias, Lucas Lopes dos Reis, Otavio Augusto Garbin de Souza, Miquel Robert Ferrer, Sérgio Luiz Carlos dos Santos, Raul Osiecki

**Affiliations:** ^1^Laboratory Federal University of Paraná- UFPR, Organization Research and Studies Group in Physical Education, Health and Performance - GPESDE/Faculty of Technology and Sciences of Northern Paraná -UNIFATECIE, Curitiba, Brazil; ^2^Laboratory University of Barcelona- UB, Faculty of Education, Department of Physical Education, Physical Activity, Physical Education and Sports, University of Barcelona, Barcelona, Spain; ^3^UNIFATECIE Laboratory, Organization of the Study and Research Group in Physical Education, Health and Performance - GPESDE/UNIFATECIE, Paranavaí, Brazil; ^4^Laboratory State University of Northern Paraná- UENP, Department Health Sciences Center- CCS, Organization Research Group on Lifestyle, Exercise and Health - GPVES/UENP, Jacarezinho, Brazil; ^5^Laboratory State University of Northern Paraná- UENP, Department Health Sciences Center- CCS, Organization Research Group on Lifestyle, Exercise and Health - GPEVES/UENP, Jacarezinho, Brazil; ^6^Laboratory University of Northern Paraná- UNOPAR, Organization Research and Studies Group in Physical Education, Health and Performance - GPESDE/Faculty of Technology and Sciences of Northern Paraná -UNIFATECIE, Londrina, Brazil

**Keywords:** burnout, emotional health, health, physical activity, students

## Abstract

Risk behaviors and signs of *burnout* are associated with substantial health losses and university dropouts. Physical activity can be an effective approach to reduce these factors. The objective of this study was to analyze aspects related to health behaviors, physical activity, and signs of *burnout* in university students and their association with physical activity. The probabilistic cluster sample consisted of 3,578 regularly enrolled undergraduate students from UFPR in Curitiba, based on a population sample of 24,032 university students. The students completed the *MBI-SS and NCHA II instruments*. Descriptive statistics were used to identify demographic indicators and characteristics of the university environment. For the proportion of subjects with respective confidence intervals (*CI* = 95%), contingency tables involving the chi-square test (χ^2^) were used. The prevalence of signs of *burnout* was estimated in punctual proportions accompanied by the respective confidence intervals (*CI* = 95%). To analyze the associations between the independent variables and signs of *burnout*, the Hierarchical Logistic Regression was used through an analysis adjusted by the other independent variables involved in the models (*CI* = 95%). Results showed that the prevalence of individuals who showed signs of *burnout* was 40.4%. The hierarchical multiple regression model pointed to: female sex (OR = 1.30; 1.11–1.51); age between 20–24 years (OR = 1.51; 1.25–1.83); and 25–29 years (OR = 1.69; 1.27–2.24); being single (OR = 2.67; 1.01–7.10); presenting regular/poor health perception (OR = 1.59; 1.13–2.22), belonging to Human Sciences courses (OR = 1.37; 1.14–1.64); attending 2nd or 3rd year (OR = 1.34; 1.12–1.61); poor academic performance (OR = 5.35; 4.11–6.96); mean (OR = 2.08; 1.78–2.43). We conclude that academics showed a high prevalence of health risk behaviors and correlate and diagnose emotional problems and signs of *burnout*. Signs of *burnout* were significantly associated with the practice of physical activity in its three dimensions; however, in the adjusted analysis for demographic indicators, the characteristics of the university environment, and health behaviors, physical activity was not significant for the model.

## Introduction

Although initially linked to the field of professional practice, studies on *burnout* have begun to investigate pre-professional scope in university students. This signals attempts to prevent the early evolution of this phenomenon, from the study phase to the labor market (Robins et al., 2018). Even if the university class is not considered to belong to the work environment, the students' activities can be interpreted as pre-professional. The workload in the academic context corresponds to the demands of studies, as they develop specific mandatory activities such as studying, facing practical classes, internships, assessment activities, competitive academic environment, generating conflicts and stress (Marôco et al., 2020).

*Burnout* has started to be investigated among university students, expanding its concept and confirming the existence of three dimensions (emotional exhaustion, disbelief, and professional/personal effectiveness), derived from the *Maslach Burnout Inventory – MBI*, also in this population (Schaufeli et al., 2002). One of the most accepted concepts reported that *burnout* is an emotional response to chronic stress situations (Guedes and Souza, 2015).

Among the types of treatment to reduce symptoms, physical activity can be an essential strategy. Naczenski et al. (2017) reported in a systematic review of 10 studies addressing the topic in the general population. Furthermore, most research involving physical activity and signs of *burnout* was carried out in different countries or with a non-university population (Lindwall et al., 2014; Olson et al., 2014), which means there is a gap in the literature.

The university population in general, has in their routine, high demand for workload and academic activities, many of these have short deadlines. In this context, it seems that university students are exposed to and show signs of mental and emotional exhaustion. One of the ways of controlling burnout is to practice physical activity. The routine practice of physical activity can bring mental relief and more willingness for the student to perform tasks. The relationship between physical activity and burnout is addressed in the present study since activity can act as a protective factor against, prevent physical symptoms, and control burnout (Bielemann et al., 2007; Batista and Ornellas, 2013).

This study aimed to assess the prevalence and associated determinants between signs of *burnout*, physical activity, and health behaviors in university students.

## Methods

### Sample Selection

The reference population included undergraduate students from the UFPR Campus, regularly enrolled in the second semester of the 2019 school year, in Curitiba, Paraná. To illustrate the dimension of the population universe to be treated, according to information from the Institution's Undergraduate Dean, 24,032 university students were enrolled at the beginning of the 2019 academic year in the 77 undergraduate courses offered on the Curitiba Campus.

The sample selection procedures followed a sequence of steps to obtain a probabilistic cluster sampling that could effectively represent the population of university students at UFPR, the year 2019. First, the classes were chosen by sampling stratified by large areas of education, namely: (a) Human Sciences (8,846); (b) Exact Sciences (9,910); (c) Biological Sciences (5,276), the prevalence of outcome (signs of *burnout*) unknown (50%). Then, the sample calculation resulted from the sum of the population of each stratum, assuming a confidence interval of 95% and a sampling error of 3.0%.

Based on the 24,032 university students enrolled in the institution's undergraduate courses, initially, the minimum number of subjects measured was 1,861 students. However, considering that the sampling design involved clusters, the effect of sampling design was added – *deff* equivalent to 1.5 and 20.0% for cases of loss, thus resulting in a predicted sample size of 3,350 university students. However, for data analysis purposes, 3,578 university students were gathered, 1,868 female and 1,710 male.

Regarding the selection of university students to compose the sample, there was a concern to obtain a representation proportional to the population considered, having as a reference for this proportionality the number of university students in the significant areas of study, course, year, and shift (morning, night, and full time) in which they were enrolled.

### Data Collection

The application of the questionnaires was carried out by two researchers. The researchers were trained in the procedures for 4 weeks at the Physical Performance Studies Center CEPFIS, and a pilot data collection was carried out with Physical Education students from UFPR 30 days before the start. The academics were gathered in a classroom, where the objectives of the research project, the principles of confidentiality, non-identification in the study, and no influence on academic performance were explained. At this time, university students were invited to participate in the study and received guidance on how to fill out the Informed Consent Form.

Subsequently, those university students who agreed to participate received questionnaires with instructions and recommendations for self-completion, with no time limit established for its completion. The questionnaires were answered individually, and any doubts expressed by the respondents were promptly clarified by the researcher monitoring the data collection.

Inclusion criteria: Students of both sexes, regularly enrolled in the second semester of 2019, present in the classroom in August, who completed and signed the TCLE. Exclusion criteria: (a) students who were unable to complete the questionnaire by the end; (b) students with some mental limitations to fill in, and those who turned in incomplete or illegible questionnaires.

### Measuring Instruments

#### Health Behavior

According to the proposed objectives for the present study, questions from the instrument were abstracted *National College Health Assessment II* in the following domains: health, health education, and safety.

In the case of the domain *health, health, and safety education*, the following question was used: “In general, how do you describe your health.” The answers were categorized according to the study by Adams et al. (2007) as (a) excellent; (b) very good; (c) good, and (d) regular and bad, and for the effect of results, the answers “I do not know” were not used.

To examine the practice of physical activity, the question “*In the last seven days, how often did you practice?”* was used. We asked whether they had undertaken, aerobic/cardiorespiratory exercise of moderate-intensity (causing a moderate increase in heart rate, such as a brisk walk), vigorous-intensity (causing a significant increase in heart and respiratory rate, such as running), and strength training (weight training at ~8–12 repetitions in each set). For the cut-off points, a study by Elliot et al. (2012) was used, which included: (a) no day; (b) 1–2 days/week; (c) 3–4 days/week; and (d) >5 days/week. We sought to identify whether the university meets the international criteria for acceptable physical activity practice (5 × 30 min/day of moderate activity, or 3 × 20 min/day of vigorous activity) as well as muscle-strengthening exercise. Therefore, a gradient was established to categorize good physical activity practice. Thus, it was unnecessary to include a new measurement instrument.

#### Demographic Indicators and Characteristics of the University Environment

The following demographic indicators were used, namely *age*: (a) < 19 years; (b) 20–24 years; (c) 25–29 years; and (d) >30 years; *the sex*: (a) men and (b) women.

As for the characteristics of the university environment, the following categories were used: *large area of study:* (a) Biological Sciences; (b) Human Sciences; (c) Exact Sciences; *study shift:* (a) morning; (b) night; and (c) full; and *academic performance:* (a) good (very good and good); (b) medium; (c) weak (weak).

#### Maslach Burnout Inventory—Student Survey—MBI-SS

To measure signs of *burnout*, the scale by *MBI-SS* Schaufeli et al. (2002) was used, self-applicable referring to the feelings/emotions of students in the university context. The questionnaire consists of 15 questions that are subdivided into three subscales: e*motional exhaustion* (5 items), *disbelief* (4 items), and *professional/personal effectiveness* (6 items). Their frequency measures all items, ranging from 0 to 6, being 0 (never), 1 (a few times a year), 2 (once a month), 3 (a few times a month), 4 (once a month). Week), 5 (a few times a week), and 6 (every day). The internal consistency of the questionnaire in the Portuguese version (2012) was assessed using Cronbach's alpha coefficient, similar to moderate to strong between scales (*r* = 0.31–0.64). Validity was measured by correlation *Pearson's* and reliability by alpha index *Cronbach's* in three different universities (Schaufeli et al., 2002).

To identify signs of *burnout* in university students, studies by Peres et al. (2014) and Viana et al. (2014) were used to determine the cut-off points. First, the distribution of responses (%) for each item of the questionnaire, *emotional exhaustion, disbelief, and professional/personal effectiveness was* calculated. Next, the mean, standard deviation, first and second tertiles of each dimension were calculated. The highest tertile is assumed to be at risk for the dimensions of *physical, emotional, and disbelief exhaustion*, and the lowest tertile for *professional/personal effectiveness*.

Thus, subjects with high signs of burnout were identified (when the three dimensions were located according to the tertiles mentioned above), subjects with moderate risk of *burnout* (when two of their dimensions were located according to the tertiles mentioned above), subject with low risk for *burnout* (when one of its three dimensions were located according to the tertiles mentioned above), finally, subjects without any risk for *burnout* (when all its dimensions were located according to the tertiles mentioned above).

For the Hierarchical Logistic Regression, there was a dichotomization of the variable, signs of *burnout* in (a) absence of signs, and (b) signs of *Burnout* (signs of *burnout* low, signs of *burnout* moderate, and signs of *burnout* high).

### Statistical Treatment

The prevalence estimates equivalent to health behaviors, the practice of moderate and vigorous cardiorespiratory/aerobic physical activity, strength training, and signs of *burnout* due to demographic indicators and health perception were presented in punctual proportions (%), accompanied by respective 95% confidence intervals (95% *CI*).

To analyze the linearity of associations between burnout signs and correlated potentials, Odds Ratio calculations were used in the SPSS 20.0 statistical program. First, statistical differences between the strata under investigation were treated by continuity correction *Yates* for 2 × 2 contingency tables, for the others, the chi-square test (χ^2^). Next, correlates that showed at least marginally significant associations (*p* ≤ 0.20) in the bivariate analysis were included in the hierarchical multiple regression procedures.

In this case, the correlates were included in blocks, with the sociodemographic variables (level 1) being the first to be included in the model, followed by those related to the characteristics of the university environment (level 2) and physical activity (level 3). Remained in the multivariate model all those related with statistical significance (*p* < 0.05).

The present study followed the ethical norms established in the Declaration of Helsinki (1975, revised in 1983). UFPR Sectors authorized the study for its execution and the Ethics and Research Involving Human Subjects of UFPR by Opinion No. 3,430.223 on July 2, 2019.

## Results

Supplementary Table 1 shows the frequency of extracts on signs of *burnout* according to sociodemographic indicators, health perception, and characterization of the university environment.

It was found that those with more signs of burnout in all classifications (low, moderate, and high) were female students with low academic performance, who perceived their health as regular/inferior. The most general category for moderate signs of burnout was enrolled in the night shift, while students with characteristic signs of burnout were between 25 and 29 years old and belonged to courses in the area of Human Sciences. Supplementary Table 2 shows the prevalence of odds ratio of signs of *burnout* with stratification for Physical Activity correlates.

This table indicates that moderate cardiorespiratory conditioning, when not practiced on any days, presents a prevalence of 46.1% and a 61% odds ratio of developing signs of burnout. The intense cardiorespiratory variable when practiced on any day of the week had a prevalence of 44.2% and an 81% odds ratio of showing signs of burnout. Strength training when practiced between 1 and 2 days/week presented a prevalence of 39.4% and a 37% odds ratio of developing signs of *burnout*, in this same variable, when not practiced any day has a 62% ratio of chance of developing *burnout*.

The associated correlates (*p* ≤ 0.20) were selected and included in the hierarchical multiple regression procedures (Supplementary Table 3), following the statistical criteria for maintaining the variables in the model. The final model consisted of the following variables: being female (*OR*: 1.30; *CI*_95%_; 1.11–1.51), aged between 20 and 24 years (*OR*: 1.51; *CI*_95%_; 1.25–1.83), and between 25 and 29 years old (*OR*: 1.69; *CI*_95%_; 1.27–2.24) and having regular/poor self-perceived health (*OR*: 1.59; *CI*_95%_; 1.13–2.22) were the first to enter the statistical model, composing the level “1.” Level “2” was composed of the variables: belonging to courses in the area of Human Sciences (*OR*: 1.37; *CI*_95%_; 1.14–1.64), having poor academic performance (*OR*: 5.35; *CI*_95%_; 4.11–6.96), and average (*OR*: 2.08; *CI*_95%_; 1.78–2.43). Level “3,” physical activity in its dimensions (cardiorespiratory/aerobic of moderate-intensity, vigorous-intensity, and strength training) were not significant in the model.

## Discussion

This study aimed to assess the prevalence and associated determinants between signs of *burnout*, physical activity, and health behaviors in university students.

Generally speaking, there are many determinants of health behaviors, however, we still lack research that addresses the topic, especially among university students. Identifying and interfering with these behaviors is of paramount importance, as this is the only way to reduce the onset, regularization, and consequences that these behaviors have inflicted on young students (Das and Horton, 2012).

The presence of signs of *burnout* in the selected samples was 40.4%, with 3.1% for signs of high *burnout*, 8.0% for signs of *burnout* moderate, and 29.3% for signs of *burnout* low. To identify signs of *burnout* in university students in this research, we used cut-off points similar to the studies by Peres et al. (2014) and Viana et al. (2014).

Exposure to burnout symptoms for a long period can cause serious damage to health, especially in mental and emotional skills. It is believed that low levels of burnout evolve in the autonomy and performance of university students, improving the ways they solve problems in their academic career. The results of this study alert the scientific community to develop goals that favor the best performance of the mind through activities that lead students to a state of relaxation and mental lightness. The results reveal that it is important to encourage them to increase the levels of physical activity they undertake each week and reflect on important psychological issues such as self-care, and awareness of a healthier lifestyle. Universities should encourage university students to set aside a specific time of the day for students to disconnect from their tasks and connect with their bodies and mind on the move.

Results regarding the presence of burnout signs in the region of the Americas were found in 41.6% of university students in Barranquilla-Colombia (Caballero et al., 2007), 55.0% of medical students in Texas- United States (Chang et al., 2012), 56.2% of students in a public university of São Paulo-Brazil (Peres et al., 2014), 65.1% of health sciences academics from Montes Claros-Brazil (Viana et al., 2014). While in the Middle East, in Saudi Arabia, 67.1% of health sciences academics showed signs of burnout (Almalki et al., 2017).

Regarding the presence of signs of *burnout, a* meta-analysis carried out by Low et al. (2019) with medical students showed a prevalence of 27.7% in European university students, 51.0% in Asian university students, and 51.6% in North American university students.

Other studies that used the *MBI-SS* as an instrument presented results for signs of *burnout that were* much lower than the results of this research. For example, signs of *burnout* were reported in 12.0% of university students in the city of Porto (Barbosa et al., 2016), 7.4% in medical students from the Sultan (Al-alawi et al., 2017), 17.0% of dentistry students in Araraquara (ENESP) (Campos and Maroco, 2012), 18.8% of nursing students in Costa Rica (Reyes and Blanco, 2016). Despite the use of the same instrument (*MBI-SS)*, differences in the prevalence of signs of *burnout* were possibly found due to the methodologies used, for example, a sample composed of first-year students only (Barbosa et al., 2016), sample size (Campos and Maroco, 2012), different cut-off points for categorizing signs of *burnout* (Reyes and Blanco, 2016).

Signs of *burnout* were found to a greater or lesser degree, as varying according to methodological heterogeneity, such as different measurement instruments, cultural differences, sample size, categorization of cut-off points, and time of application of the research. Significant associations with signs of *burnout* through the chi-square strata (χ^2^) were found for female students, aged between 25 and 29 years, from the night study period, belonging to the Human Sciences area, with weak academic performance and inadequate/regular self-perception of health.

In the prevalence analysis through the *odds ratio* of signs *burnout* with stratification for physical activity correlates, fewer days of practice were associated with greater chances of signs *of burnout*, especially for those who did not practice physical activity in any of the dimensions moderate-intensity cardiorespiratory/aerobic, vigorous-intensity, and strength training on any day of the week.

Corroborating the findings of this research, physical activity was significantly associated with lower signs of *burnout* in several studies (Weight et al., 2013; Cecil et al., 2014; Lindwall et al., 2014; Olson et al., 2014; Fares et al., 2015; Farias et al., 2019). To explain such relationships, psychological mechanisms were reported as a way to reduce chronic stress and, consequently, signs of *burnout* (Sonnentag, 2012), either through increased self-efficacy (Joseph et al., 2014), through increases in sense of competence to deal with tasks (Feuerhahn et al., 2014) or make them less demanding (Hockey, 2013).

About physiological mechanisms, physical activity may be able to improve the relationship with psychological stress (cardiovascular fitness hypothesis), causing a more significant recovery of the body to stress exposure factors (Klaperski et al., 2014), inducing changes in several neurotransmitters and neuromodulators, with consequent improvement in mood and increased energy (Schuch et al., 2016).

Questions related to physical activity in its dimensions required the respondent to remember the practice in the previous 7 days, while questions related to signs of *burnout* assessed the prevalence in the last year. This is worrisome given that it might not be possible to determine when signs of *burnout* were established during the previous year, nor to determine whether the practice of reported physical activity reflected a pre-existing behavior. Thus, the validity of these findings depends on the assumption that physical activity indicates previous behavior. However, careful consideration of alternative possibilities is necessary (Naczenski et al., 2017). When adjusted for the variables of demographic indicators, the characteristics of the university environment, and correlates of health behaviors, physical activity in its dimensions was not significant for the model.

The absence of an association between signs of *burnout* and physical activity in the adjusted model may have been because, despite the high prevalence of 40.4% for “signs of *burnout*,” the prevalence of college students with signs of *burnout* was only 3.1%. This is a high result for the dimensions “emotional exhaustion” and “disbelief,” and a low result for “*professional/personal effectiveness”* through the categorization of tertiles.

Taking into account the hypothesis that *burnout* develops in the form of a continuum with the beginning of the process possibly occurring in the emotional exhaustion dimension (Guedes and Souza, 2015), it is assumed that a good part of the almost 29.3% of students who showed signs of *burnout* as being low, presented results only for the dimension *emotional exhaustion*.

This justification is supported in the literature through the results of a systematic review carried out by Naczenski et al. (2017) in the general population, where there was a negative relationship between physical activity and only the dimension and *emotional exhaustion* of *burnout* (Bretland and Thorsteinsson, 2015; Lindegard et al., 2015). In the few studies that investigated the dimensions of *disbelief* and *low effectiveness* professional/*personal*, the evidence was inconsistent (Gerber et al., 2013; Freitas et al., 2014; Bretland and Thorsteinsson, 2015).

Thus, the hypothesis that physical activity is a practical approach to reduce *burnout was* not confirmed in the present study after the adjusted regression model, thus lacking further investigation, primarily through intervention studies and research that assess the association of physical activity with each dimension of *burnout*, separately. These statements are justified by the fact that in this same review by Naczenski et al. (2017) with the general population, this research model proved to be more efficient, demonstrating association results with the dimension *emotional exhaustion* more consistently (Van Rhenen et al., 2005; Gerber et al., 2013; Tsai et al., 2013; Freitas et al., 2014; Bretland and Thorsteinsson, 2015; Lindegard et al., 2015).

More prospectively designed research studies would help to determine whether the practice of physical activity, in all its dimensions, is causally related to lower signs of *burnout*. In addition, such studies could more fully justify the practice of physical activity as a therapeutic complement to alleviate *burnout*.

Most questions relating to this subject relate to the time recall type and college students should not be expected to accurately recall behaviors from the previous year. Regarding physical activity, the measures of practice in its dimensions were based on weekly frequency recall questions, but the volume, intensity, or consistency of the practice of these exercises was not verified. Finally, cross-sectional data such as that used in the present research allows for the construction of cross-sectional association models, but prevents the assessment of causality. However, with the collected data, it is possible to have a parameter of the importance of the weekly physical activity to control burnout signs in university students. In addition, the research was carried out in an important university environment of a federal institution and had a very representative sample size, which brings reliability to the results. Through this cross-sectional model association, it is possible to create a study design that evaluates the sample longitudinally, which will give greater consistency to the investigated theme.

## Conclusions

Despite the high prevalence of signs of *burnout* (low, moderate, and high), signs of *burnout* high were reported by only a tiny portion of the selected sample. After adjusted regression analysis, female students, aged between 20 and 29 years, who were married, with poor academic performance and very high stress, deserve special attention.

In the crude analysis, signs of *burnout* were significantly associated with the practice of physical activity in its three dimensions; however, in the analysis adjusted for demographic indicators, the characteristics of the university environment, and health behaviors and physical activity were found to be non-significant for the model.

## Data Availability Statement

The raw data supporting the conclusions of this article will be made available by the authors, without undue reservation.

## Ethics Statement

The studies involving human participants were reviewed and approved by Ethics and Research Committee Involving Human Beings at UFPR through Opinion No. 3,430.223 on 07/02/2019. The patients/participants provided their written informed consent to participate in this study.

## Author Contributions

RS designed the study. RS, FR, RE, LR, OG, RO, MR, and SS participated in the project and coordination and wrote the manuscript. All authors read and approved the final version of the manuscript and agreed with the authors' order of presentation.

## Conflict of Interest

The authors declare that the research was conducted in the absence of any commercial or financial relationships that could be construed as a potential conflict of interest.

## Publisher's Note

All claims expressed in this article are solely those of the authors and do not necessarily represent those of their affiliated organizations, or those of the publisher, the editors and the reviewers. Any product that may be evaluated in this article, or claim that may be made by its manufacturer, is not guaranteed or endorsed by the publisher.
