# Supplementary material for: Associated Determinants Between Evidence of Burnout, Physical Activity, and Health Behaviors of University Students
Source: Front Sports Act Living. 2021 Oct 20;3:733309. doi: 10.3389/fspor.2021.733309 (PMC8568456; doi:10.3389/fspor.2021.733309)
Supplement: Supplementary file 1 [file Data_Sheet_1.PDF]

Supplementary Material

Supplementary Tables

Table 01 - Frequency of extracts on evidence *burnout* according to sociodemographic indicators, perception of Health, and characteristics of the university environment (n = 3.578).

**Demographic Indicators and University Environment**	***Indications of Burnout***			
	Absence of indications of *Burnout*	Indications of *Burnout* Low	Indications of *Burnout* moderate	Indications of *Burnout* high
	Total (95%*CI*)	Total (95%*CI*)	Total (95%*CI*)	Total (95%*CI*)
**Total**	59.6 (57.9 - 61.2)	29.3 (27.8 - 30.8)	8.0 (7.2 - 8.9)	3.1 (2.6 - 3.7)
**Gender**	$\chi2$ = 10.971<br>p < 0.001	$\chi2$ = 5.219<br>p = 0.022	$\chi2$ = 9.575<br>p = 0.002	$\chi2$ = 7.561<br>p = 0.006
Male (n = 1,709)	64.1 (61.8 -66.3)	27.2 (25.1 - 29)	.36.5 (5.4 - 7.7)	2.3 (1.6 - 3.1)
Female (n = 1,869)	55.4 (53.2 - 57.7)	31.2 (29 .2 - 33.4)	9.4 (8.1 - 10.8)	3.9 (3.1 - 4.8)
**Age**	$\chi2$ = 9.688<br>p = 0.021	$\chi2$ = 7.979<br>p = 0.046	$\chi2$ = 5.050<br>p = 0.168	$\chi2$ = 4.669<br>p = 0.198
≤ 19 years (n = 1,043)	65.0 (62.1 - 67.9)	25.6 (23.0 - 28.3)	6.7 (5.3 - 8.3)	2.7 (1.8 - 3.8)
20 - 24 years (n = 1,942)	56.8 (54.6 - 59.0)	30.8 (28.8 - 32.9)	8.9 (7 .6 - 10.2)	3.5 (2.7 - 4.4)
25 - 29 years (n = 393)	55.7 (50.8 - 60.6)	32.3 (27.8 -37, 0)	8.4 (5.9 - 11.4)	3.6 (2.0 -5.7)

≥ 30 years (n = 200)	65.5 (58.7 - 71, 9)	27.5 (21.6 - 33.9)	6.0 (3.3 - 9.9)	1.0 (0.2 - 3.1)
**Areas**	χ2 = 4.841<br>p = 0.089	χ2 = 11.025<br>p = 0.004	χ2 = 0.163<br>p = 0.922	χ2 = 0.799<br>p = 0.671
Exact (n = 1,264)	60.0 (57.3 - 62.7)	28.2 (25.8 - 30.8)	8.2 (6.8 - 9.8)	3.5 (2.6 - 4.6)
Biological (n = 1,217)	62.6 (59.9 - 65.3)	26.5 (24.0 - 29.0)	8.1 (6.6 - 9.7)	2.9 (2.0 - 3.9)
Human (n = 1,096)	55.6 (52.6 - 58.5)	33.7 (30.9 - 36.5)	7.8 (6.3 - 9.4)	3.0 (2.1 - 4.1)
**Shift**	χ2 = 7.263<br>p = 0.026	χ2 = 6.218<br>p = 0.045	χ2 = 9.796<br>p = 0.007	χ2 = 0.435<br>p = 0.804
Daytime (n = 1,655)	62.9 (60.6 - 65.2)	27.6 (25.4 - 29.7)	6.5 (5.3 - 7.7)	3.1 (2.3 - 4.0)
Night (n = 802)	54.0 (50.5 - 57.4)	33.3 (30.1 - 36.6)	9.9 (7.9 - 12.0)	2.9 (1.9 - 4.2)
Integral (n = 1.121)	58.6 (55.7 - 61.5)	29.0 (26.4 - 31.7)	9.0 (7.4 - 10.8)	3.4 (2.4 - 4.6)
**Academic Achievement**	χ2 = 121.480<br>p <0.001	χ2 = 65.637<br>p <0.001	χ2 = 98.987<br>p <0.001	χ2 = 102.251<br>p <0.001
Good (n = 1881)	71.2 (69, 2 - 73.3)	22.5 (20.6 - 24.4)	4.7 (3.8 -5.8)	1.5 (1.0 - 2.2)
Average (n = 1,318)	52, 2 (49.5 - 5 4.9)	35.7 (33.1 - 38.3)	9.2 (7.7 - 10.8)	3.0 (2.1 - 4.0)
Weak (n = 379)	27.2 (22.9 - 31.8)	40.9 (36.0 - 45.9)	20.3 (16.5 - 24.6)	11.6 (8.6 - 15.1)

**Perception of Health**	$\chi2 = 68.123$<br>$p < 0.001$	$\chi2 = 33.705$<br>$p < 0.001$	$\chi2 = 59.392$<br>$p < 0.001$	$\chi2 = 53.361$<br>$p < 0.001$
Excellent (n = 260)	69.6 (63.9 - 75.0)	21.9 (17.2 - 27.2)	7.3 (4.6 - 10.9)	1.2 (0.3 - 3.0)
Very Good (n = 1,012)	71.7 (68.9 - 74.5)	22.8 (20, 3 - 25.5)	4.2 (3.1 - 5.6)	1.2 (0.6 - 2.0)
Good (n = 1.497)	58.7 (56.1 - 61.1)	31, 3 (29.0 - 33.7)	7.3 (6.0 - 8.7)	2.7 (2.0 - 3.6)
Fair / Poor (n = 809)	42.8 (39.4 - 46.2)	36.0 (32.7 - 39.3)	14.3 (12.0 - 16.9)	6.9 (5.3 - 8.8)

Table 02 - Prevalence (95% *CI*) and *odds ratio* (95% *CI*) of signs of *Burnout* with stratification for related physical activity (n = 3.578).

**Physical Activity**	**Indications of *Burnout***		
	**Prevalence (95% *CI*)**	**OR crude (95% *CI*)**	***p*-value**
Moderate Cardiorespiratory			
≥ Five days/week	34.7 (30.8 - 38.8)	Reference	< 0.001
3 - 4 days / week	36.6 (33.0 - 40.3)	1.09 (0.86 - 1.38)	
1 - 2 days / week	38.2 (35.2 - 41.2)	1.16 (0.93 - 1.45)	
No day	46.1 (43.5 - 48.7)	1.61 (1, 31 - 1.98)	
Intense Cardiorespiratory			
≥ Five days/week	30.4 (24.7 - 36.6)	Reference	< 0.001

3 - 4 days / week	34.9 (30.1 - 39.8)	1.23 (0.86 - 1.75)	
1 - 2 days / week	36.7 (33.6 - 39.9)	1.33 (0.97 - 1.82)	
No day	44.2 (42.0 - 46.3)	1.81 (1, 35 - 2.43)	
Strength Training			
≥ Five days/week	32.1 (27.6 - 36.9)	Reference	
3 - 4 days / week	34.6 (30.4 - 39.0)	1.12 (0.84 - 1.49)	< 0.001
1 - 2 days / week	39.4 (35.2 - 43.7)	1.37 (1.04 - 1.81)	
No day	43.4 (41.3 - 45.4)	1.62 (1, 29 - 2.03)	

SOURCE: The author (2020). CAPTION: *OR: Odds Ratio.*

Table 03 - Multiple hierarchical logistics regression for sociodemographic correlates and health perception (level 1), university environment (level 2), and physical activity (level 3) evidence of *Burnout* (n = 3.578).

	**Indications of *Burnout***	
**Independent Variables**	**OR adjusted (95% *CI*)**	p-value
**<u>Level 1</u>**		
**Sex**		
Male	Reference	0.001
Female	1.30 (1.11 - 1.51)	
Age		

≤ 19 years	Reference	
20-24 years	1.51 (1.25 - 1.83)	< 0.001
25-29 years	1.69 (1.27 - 2.24)	
≥ 30 years	1.25 (0.85 - 1 .85)	
**Health Perception**		
Excellent	Reference	
Very good	0.82 (0.60 - 1.14)	< 0.001
Good	1.11 (0.81 - 1.52)	
Fair / Poor	1.59 (1.13 - 2.22)	
**<u>Level 2</u>**		
**Big area**		
Exact	Reference	
Biological	0.96 (0.80 - 1.16)	<0.001
Human	1.37 (1.14 - 1.64)	
**Academic Performance**		
Good	Reference	
Medium	2.08 (1.78 - 2.43)	< 0.001
Poor	5.35 (4.11 - 6.96)	
**Level 3 -** Not significant in the model		

SOURCE: The author. CAPTION: *OR: Odds Ratio.*
